# Complaints linked to vestibular dysfunction are associated with tau pathology and hippocampal volume

**DOI:** 10.1002/alz70857_102137

**Published:** 2025-12-24

**Authors:** Avinash Chandra, Sheena Waters, Yue Liu, Viktoria Azoidou, Charles R Marshall, Laura J Smith

**Affiliations:** ^1^ Queen Mary University of London, London, Greater London, United Kingdom

## Abstract

**Background:**

Increased dementia risk and cognitive decline are linked to impairments in the vestibular system, which comprises of peripheral structures and cortical projections, and is responsible maintaining for balance and posture. Subjective dizziness, in addition to several other symptoms, is often a consequence of vestibular dysfunction or damage. However, the neuropathological underpinning for how this clinical phenotype may impact cognition remains unclear.

**Method:**

Data was included for 1677 participants in the Alzheimer's Disease Neuroimaging Initiative with self‐reported medical history, demographics and cognitive performance. Subsets of patients underwent assessment with *in vivo* biomarkers including cerebrospinal fluid (CSF) Alzheimer's disease (AD) markers or MRI/PET scanning. Regression models were used to examine associations between (1) self‐reported dizziness alone and (2) self‐reported dizziness combined with at least one additional symptom of vestibular dysfunction, with clinical, cognitive, and biomarker outcomes. This approach was refined to better identify cases potentially affected by vestibular‐related impairments.

**Result:**

In cognitively normal (CN) subjects, dizziness and at least one other symptom of vestibular dysfunction was associated with cerebrospinal fluid (CSF) t‐tau levels (β=0.41; 95% CI: 0.07 to 0.79; *p* = 0.025). Moreover, for CN subjects, self‐reported dizziness was associated with lower hippocampal volume (β = –0.05; 95% CI: –0.09 to – 0.009; *p* = 0.017). These associations were demonstrated at least at the trend level in combined groups of cognitively normal, mild cognitive impairment (MCI), and AD cases. No similar associations were found for MCI or AD risk, cognitive performance, amyloid‐β deposition measured on PET or CSF, CSF *p*‐tau, total brain volume, or white matter hyperintensities.

**Conclusion:**

This study provides new evidence shedding light on early degenerative markers that may potentially be linked to vestibular dysfunction. Tau pathology and hippocampal atrophy may be a cause or consequence of damage to the vestibular system; however, future is needed to clarify the direction of this relationship. Consideration of vestibular dysfunction may also be warranted when clinically evaluating the basis on early‐stage neurodegenerative processes.

